# Controlling hypoxia-inducible factor-2α is critical for maintaining bone homeostasis in mice

**DOI:** 10.1038/s41413-019-0054-y

**Published:** 2019-05-13

**Authors:** Sun Young Lee, Ka Hyon Park, Hyung-Gu Yu, Eunbyul Kook, Won-Hyun Song, Gyuseok Lee, Jeong-Tae Koh, Hong-In Shin, Je-Yong Choi, Yun Hyun Huh, Je-Hwang Ryu

**Affiliations:** 1 Department of Pharmacology and Dental Therapeutics, School of Dentistry, Chonnam National University, Gwangju, 61186 Korea; 2 Research Center for Biomineralization Disorders, School of Dentistry, Chonnam National University, Gwangju, 61186 Korea; 3 Department of Oral Pathology and IHBR, School of Dentistry, Kyungpook National University, Daegu, 41940 Korea; 4 Department of Biochemistry and Cell Biology, BK21 Plus KNU Biomedical Convergence Program, Skeletal Diseases Analysis Center, Korea Mouse Phenotyping Center (KMPC), School of Medicine, Kyungpook National University, Daegu, 41944 Korea; 5 School of Life Sciences, Gwangju Institute of Science and Technology, Gwangju, 61005 Korea

**Keywords:** Diseases, Bone

## Abstract

Pathological bone loss is caused by an imbalance between bone formation and resorption. The bone microenvironments are hypoxic, and hypoxia-inducible factor (HIF) is known to play notable roles in bone remodeling. However, the relevant functions of HIF-2α are not well understood. Here, we have shown that HIF-2α deficiency in mice enhances bone mass through its effects on the differentiation of osteoblasts and osteoclasts. In vitro analyses revealed that HIF-2α inhibits osteoblast differentiation by targeting *Twist2* and stimulates RANKL-induced osteoclastogenesis via regulation of *Traf6*. In addition, HIF-2α appears to contribute to the crosstalk between osteoblasts and osteoclasts by directly targeting RANKL in osteoprogenitor cells. Experiments performed with osteoblast- and osteoclast-specific conditional knockout mice supported a role of HIF-2α in this crosstalk. HIF-2α deficiency alleviated ovariectomy-induced bone loss in mice, and specific inhibition of HIF-2α with ZINC04179524 significantly blocked RANKL-mediated osteoclastogenesis. Collectively, our results suggest that HIF-2α functions as a catabolic regulator in bone remodeling, which is critical for the maintenance of bone homeostasis.

## Introduction

Bone mass is maintained by continuous bone remodeling, wherein preexisting bone is broken down by osteoclasts and rebuilt by osteoblasts^1^. The balance between osteoclastic and osteoblastic activities is coordinately regulated by an interplay between the two cell types^1–3^. Dysregulation of bone homeostasis causes pathophysiological bone diseases such as osteoporosis, which is the most common metabolic bone disease^4,5^. This chronic disease of multifactorial etiology is characterized by a decrease in the density of bone. Studies seeking to identify novel regulators of bone homeostasis are very important in the efforts to understand and treat diseases associated with bone remodeling or osteoporotic bone loss.

The differentiation of osteoblasts from mesenchymal stem cells, which is an important step in bone formation, is controlled by RUNX2-mediated (runt-related transcription factor 2) and osterix-mediated transcriptional regulation^6,7^. On the other hand, osteoclasts are multinucleated giant cells that arise from hematopoietic progenitors of the monocyte-macrophage lineage; they develop through a sequence of steps that include an initial proliferation phase, a later differentiation phase and maturation^8,9^. Macrophage colony-stimulating factor (M-CSF) is indispensable for the proliferation of preosteoclasts, and receptor activator of NF-κB ligand (RANKL) plays a pivotal role in the differentiation and maturation of osteoclasts^10–12^. Several soluble factors and signaling pathways, including RANK, its ligand, RANKL^11^, ephrinB2/EphB4^12^, Sema4D^13^, and Sema3A^14^, contribute to the crosstalk between osteoblasts and osteoclasts. Among these factors and pathways, the regulatory mechanism by which RANKL and osteoprotegerin (OPG) control osteoclast development and function is the most well established^15^. Osteoblast-lineage cell-derived RANKL stimulates its receptor, RANK, on osteoclast precursors to activate downstream transcription factors, such as NF-κB^16^, c-Fos^17^, and nuclear factor of activated T cells c1 (NFATc1)^18,19^, resulting in osteoclast differentiation and maturation. OPG, which is a soluble decoy receptor for RANKL, is also produced by osteoblasts and acts to balance osteoclastogenesis in vivo ^20^.

The bone microenvironments, such as the endosteal zones of bone marrow cavities and the epiphyseal growth plates, are particularly hypoxic, and hypoxia-inducible factor (HIF) is believed to contribute to the functions of chondrocytes and osteoblasts, respectively, in these areas^21,22^. HIF, which is a heterodimeric transcription factor that consists of an oxygen-regulated α subunit and a constitutively expressed β subunit, acts as a master regulator of the adaptive response to hypoxia ^23^.

Hypoxia stimulates angiogenesis-dependent osteogenesis during bone ossification, mainly via VEGF-dependent promotion of bone vascularization through HIF-1α signaling^24^. Earlier reports have shown that stabilization of HIF-1α and HIF-2α via osteoblast-specific deletion of the von Hippel–Lindau (*Vhl*) gene enhances osteogenesis by increasing vascularization and endochondral ossification through VEGF activation^22,24^. A marked decrease in trabecular bone volume has been reported in mice lacking HIF-1α. In contrast to the anabolic role of HIF-1α in bone modeling (ossification), several other studies have demonstrated that hypoxia and HIF-1α promote osteoclastogenesis and subsequent bone resorption during bone remodeling (metabolism). For instance, HIF-1α overexpression blocks BMP-2-induced osteoblast differentiation and promotes osteoclastogenesis^25^. HIF-1α accumulates in the osteoclasts of ovariectomized (OVX) estrogen-deficient female mice^26^ and orchidectomized (ORX) testosterone-deficient male mice^27^, and HIF-1α inhibition alleviates the osteoporosis normally observed in OVX or ORX mice^26–28^. The reasons underlying discrepancies regarding the roles of HIF-1α in bone formation and remodeling are unclear but may reflect systemic effects on the multiple cell types found within the bone microenvironment in vivo.

In contrast to HIF-1α, HIF-2α is insufficient to regulate angiogenesis–osteogenesis coupling and osteoblast functions during the process of new bone formation^29^. HIF-2α deficiency leads to a transient delay of endochondral bone ossification, which is attributable to effects on hypertrophic chondrocytes, not bone cells^30^. Although hypoxia and HIF are increasingly being accepted as playing critical roles in bone biology, HIF-2α, which is closely related to HIF-1α, has not been well investigated in the contexts of bone remodeling and metabolism. There are many similarities between the two isoforms, but HIF-1α and HIF-2α display distinct cellular activities and show different sensitivities to oxygen tension^23,31^. We recently reported that HIF-2α depletion downregulates RANKL expression in the fibroblast-like synoviocytes of the rheumatoid arthritis (RA) synovium^32^, suggesting that HIF-2α plays a catabolic function in bone remodeling. Here, we used constitutive *Hif-2α* heterozygous knockout (KO) mice and conditional mice with osteoblast- or osteoclast-specific depletion of HIF-2α to extensively study the functions of HIF-2α in regulating osteoblast and osteoclast differentiation during bone remodeling and in influencing the interplay between these cell types.

## Results

### Heterozygous *Hif-2α*-KO mice show increased bone mass

**Fig. 1:**
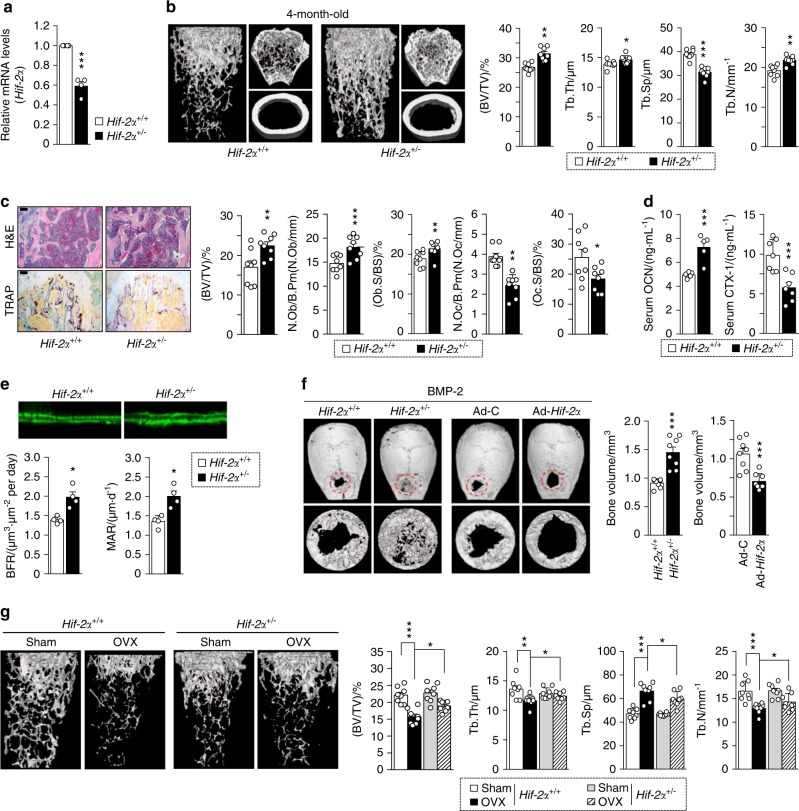
Heterozygous *Hif-2α* KO mice show increased bone mass. **a**–**e** Analysis of femoral trabecular or calvarial bones from 4-month-old *Hif-2α*^+/−^ and WT mice. mRNA levels of *Hif-2α* in femoral bone from WT (*Hif-2α*^+/+^) and *Hif-2α*^+/−^ mice (*n* = 4; **a**). Representative images of µCT reconstructions of femoral trabecular and cortical bones (**b**) and H&E and TRAP staining of trabecular bones (scale bar: 100 μm; **c**). Bone volume per tissue volume (BV/TV), trabecular bone thickness (Tb.Th), trabecular separation (Tb.Sp), and trabecular number (Tb.N) were analyzed based on the µCT measurements (*n* = 8; **b**). BV/TV, the number of osteoblastic cells per bone perimeter (N.Ob/B.Pm), the osteoblast surface normalized by bone surface (Ob.S/BS), the number of osteoclastic cells per bone perimeter (N.Oc/B.Pm), and the osteoclast surface normalized by bone surface (Oc.S/BS) were assessed by bone histomorphometric analyses of the metaphyseal regions of femurs (*n* = 8; **c**). ELISA-based measurement of the serum concentrations of OCN (*n* = 5) and CTX-1 (*n* = 7) (**d**). The bone forming rate (BFR) and mineral apposition rate (MAR) were analyzed from measurements obtained using calcein double labeling of femurs (*n* = 4; **e**). **f** Representative µCT images and measurements of bone volume of calvarial defect models generated in *Hif-2α*^+/−^ and WT mice and in C57BL/6 mice infected with Ad-*Hif-2α* or Ad-control (Ad-C) (*n* = 8). **g** Representative images of µCT reconstructions of femoral trabecular bones are shown, and quantitative µCT analysis was used to measure the BV/TV, Tb.Th, Tb.Sp, and Tb.N of the femoral bones from OVX- or sham-operated mice (*n* = 8). Values are presented as the mean ± SEM (*P < 0.05, **P < 0.01, and ***P < 0.005). The effects of OVX and genetic deletion of *Hif-2α* as well as their interaction in mice were analyzed by two-way ANOVA (**g** BV/TV: interaction = 0.048 7, OVX < 0.000 1, genetic deletion of *Hif-2α* = 0.024 5)

To determine the regulatory function of HIF-2α in bone metabolism, we first examined the bone microarchitecture of 4-month-old heterozygous *Hif-2α-*KO (*Hif-2α*^+/−^) and wild-type (WT, *Hif-2α*^+/+^) mice using X-ray microcomputed tomography (μCT). The HIF-2α transcript level was markedly lower in the femoral bone of the *Hif-2α*^+/−^ mice than in their WT littermates (Fig. 1a). The μCT images showed the presence of increased cancellous trabeculae in the *Hif-2α*^+/−^ mice (Fig. 1b). Quantitative analyses revealed that the bone volume per tissue volume (BV/TV), trabecular thickness (Tb.Th), and trabecular number (Tb.N) were greater and the trabecular separation (Tb.Sp) was smaller in heterozygous *Hif-2α*-KO mice than in WT littermates (Fig. 1b). H&E and tartrate-resistant acid phosphatase (TRAP) staining revealed that *Hif-2α*^+/−^ mice exhibited an increase in the bone trabecular percentage and a decrease in the number of osteoclasts (Fig. 1c). Bone histomorphometric analyses revealed that the BV/TV, the number of osteoblasts per bone perimeter (N.Ob/B.Pm) and the osteoblast surface per bone surface (Ob.S/BS) were greater in the *Hif-2α*^*+/−*^ mice, whereas parameters associated with bone resorption, such as the number of osteoclasts per bone perimeter (N.Oc/B.Pm) and the osteoclast surface per bone surface (Oc.S/BS) were lower in the *Hif-2α*^*+/−*^ mice (Fig. 1c). To evaluate dynamic bone formation, biochemical markers of bone turnover were measured in serum, and bone formation was visualized via calcein labeling in the femoral bone. Serum osteocalcin (OCN), a marker of bone formation, was elevated, while the serum level of the bone resorption-specific biomarker, C-terminal telopeptide (CTX)-1, was lower in the *Hif-2α*-deficient mice than in their WT counterparts (Fig. 1d). To further establish the impact of HIF-2α depletion on the dynamically assessed mineral acquisition rate, we labeled *Hif-2α*^*+/−*^ and WT mice with calcein 10, 3 days before sacrifice. Fluorochrome labeling showed that there were significant increases in the distance between the calcein-labeled surfaces and the histomorphometric parameters of bone formation rate and mineral apposition rate in the *Hif-2α*^*+/−*^ mice versus those in the WT mice (Fig. 1e). To further confirm the effects of HIF-2α on bone formation, we generated critical-size calvarial defect models using *Hif-2α*^*+/−*^ and WT mice. We found that BMP-2-induced bone regeneration was enhanced in *Hif-2α*^*+/−*^ mice and that adenoviral infection with Ad-*Hif-2α* delayed the BMP-2-induced regeneration of calvarial defects (Fig. 1f). Next, we examined the bone structure of OVX mice (Fig. 1g). Estrogen deficiency in postmenopausal females leads to an imbalance between bone formation and resorption, subsequently resulting in net bone loss and osteoporosis^33^. Although, unexpectedly, sham-operated female mice had no significant changes in bone mass resulting from HIF-2α deficiency, and OVX-induced bone loss was alleviated in *Hif-2α*^*+/−*^ mice compared to that in the WT littermates, as determined by μCT imaging and analyses of quantitative parameters, such as BV/TV, Tb.Th, Tb.Sp, and Tb.N (Fig. 1g). No differences in cortical thickness and only a modest increase in cortical volume were detected in all experimental *Hif-2α*^*+/−*^ mice (Supplementary Fig. 1). HIF-2α appeared to have a more significant effect on trabecular bone than on cortical bone. Moreover, the turnover of trabecular bone was higher than that of cortical bone during age-related changes in skeletal mass and osteoporotic bone loss^34–36^. In view of these findings, we focused on trabecular bone physiology. In addition, inconsistent with the results obtained using 4-month-old mice (Fig. 1), no significant changes in bone mass were observed in younger (4- or 8-week-old) mice (Supplementary Fig. 2). Although the reason underlying these differences has yet to be established, our findings suggest that HIF-2α contributes to the bone remodeling (metabolism) process of mature mice to a greater extent than the bone modeling (ossification) of growing young mice. Taken together, these data indicate that HIF-2α depletion may lead to increased bone mass through its effects on both osteoblasts and osteoclasts during the bone remodeling process.

### HIF-2α blocks osteoblast differentiation via TWIST2-mediated OCN regulation

**Fig. 2:**
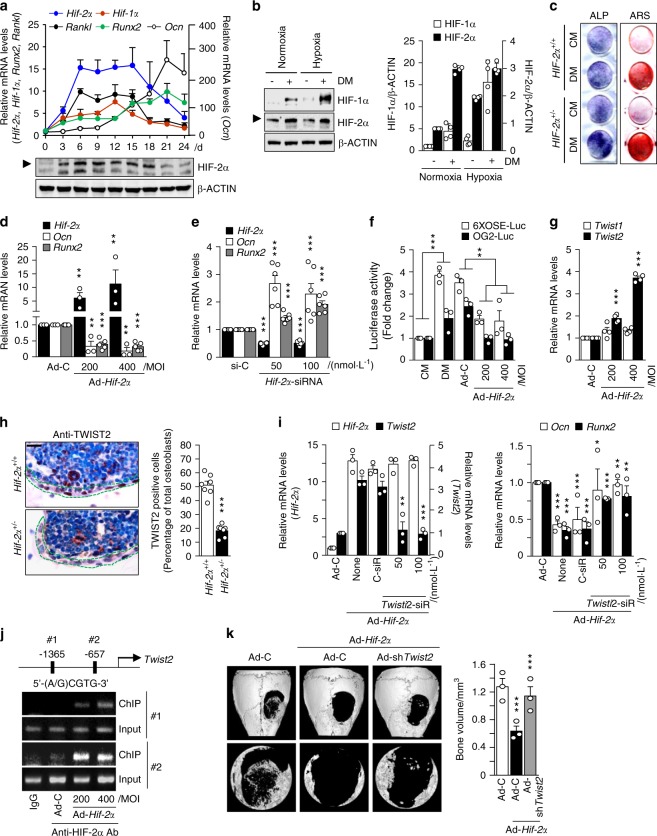
HIF-2α blocks osteoblast differentiation by inhibiting osteocalcin expression. **a** Primary calvarial preosteoblasts from WT mice were cultured in osteogenic differentiation medium containing 50 μg·mL^−1^ L-AA and 5 μmol·L^−1^ β-GP for 24 days. The transcript and protein levels of HIF-2α on the indicated culture days were determined by qRT-PCR and western blotting, respectively. The expression levels of *Hif-2α, Ocn, Runx2, Rankl*, and *Hif-1α* were analyzed by qRT-PCR (*n* = 3). **b** Western blotting and quantification of protein levels of HIF-1α and HIF-2α in undifferentiated or differentiated osteoblasts under normoxia or hypoxia (*n* = 4). DM, differentiation media. **c** Alkaline phosphatase (ALP) and alizarin red S (ARS) staining in primary calvarial preosteoblasts cultured in control media (CM) or differentiation media (DM). Calvarial preosteoblasts were obtained from WT or *Hif-2α*^+/−^ mice (*n* = 3). **d** Transcript levels of *Hif-2α*, *Ocn*, and *Runx2* were detected by qRT-PCR in primary cultured calvarial preosteoblasts infected with 400 multiplicity of infection (MOI) of Ad-C or the indicated MOI of Ad*-Hif-2α* (*n* > 3). **e** Detection of the indicated mRNAs by qRT-PCR in osteoblasts transfected with control siRNA (si-C) or the indicated amounts (nM) of *Hif-2α*-siRNA (*n* = 6). **f** RUNX2-responsive luciferase reporters (6xOSE-luc or OG2-luc) were transfected into primary calvarial preosteoblasts infected with Ad-*Hif-2α* or Ad-C. Luciferase assays were performed, and the data are presented as fold changes relative to each CM group (*n* = 3). **g** qRT-PCR analysis of *Twist1* and *Twist2* (*n* = 4). **h** TWIST2 immunostaining in osteoblasts of bone tissue from WT and *Hif-2α*^+/−^ mice. Dotted lines indicate osteoblasts (scale bar: 10 μm). Quantification of TWIST2-positive osteoblasts is shown (*n* = 7). **i** Detection of the mRNA levels of *Hif-2α, Twist2, Runx2*, and *Ocn* following the siRNA-mediated silencing of *Twist2* (*Twist2*-siR) in HIF-2α-overexpressing cells (*n* = 3). **j** ChIP assays were performed using primer pairs (1 and 2) designed to span the putative HIF-2α binding sites within the *Twist2* promoter, along with an anti-HIF-2α antibody. **k** Representative µCT images and measurements of bone volume of calvarial defect models infected with Ad-C or Ad-*Hif-2α* and coinjected with adenovirus encoding *Twist2* shRNA (Ad-sh*Twist2*) (*n* = 3). Values are presented as the mean ± SEM (*P < 0.05, **P < 0.01, and ***P < 0.005)

Because heterozygous *Hif-2α-*KO mice showed an increase in bone mass, as shown in Fig. 1, we hypothesized that downregulation of HIF-2α would modulate osteoblast differentiation. To test this hypothesis, we examined HIF-2α expression during the ascorbic acid (AA)- and β-glycerophosphate (β-Gp)-induced differentiation of preosteoblasts isolated from mouse calvaria. The transcript and protein levels of HIF-2α were markedly upregulated during osteoblast differentiation, as evidenced by the expression of osteoblast-related markers, including *Ocn*, *Runx2*, and *Rankl* (Fig. 2a). In addition, *Hif-1α* expression also increased slightly during osteoblast differentiation (Fig. 2a). The upregulation and nuclear localization of HIF-2α in preosteoblasts cultured in differentiation media containing AA and β-Gp were assessed using western blot (Fig. 2b and Supplementary Fig. 3a), and the results were confirmed with immunofluorescence staining (Supplementary Fig. 3b). The α subunits of HIF are hydroxylated at conserved proline residues to allow for proteasomal degradation under normoxia. Under hypoxic conditions, prolyl hydroxylase (PHD), which utilizes oxygen as a cosubstrate, is inhibited^37^. Accordingly, the protein levels of HIF-1α and HIF-2α were measured under hypoxic and normoxic conditions. HIF-1α protein expression was markedly upregulated during osteoblastogenesis under hypoxia, whereas HIF-2α was upregulated in differentiated osteoblasts under both conditions (Fig. 2b). The overexpression of HIF-2α mediated by infection with Ad-*Hif-2α* inhibited the AA/β-Gp-mediated osteoblast differentiation of mouse calvarial preosteoblasts, as determined by alkaline phosphatase and alizarin red S staining (Supplementary Fig. 3c), whereas increased osteoblast differentiation from *Hif-2α*^+/−^ calvarial preosteoblasts was observed (Fig. 2c). The inhibitory effect of HIF-2α overexpression on osteoblast differentiation was confirmed via the determination of the expression of osteoblast-differentiation marker genes, such as *Ocn* and *Runx2* (Fig. 2d and Supplementary Fig. 3d). Conversely, knocking down HIF-2α using a specific siRNA led to the upregulation of *Ocn* and *Runx2* (Fig. 2e and Supplementary Fig. 3e). Because RUNX2 is a well-known transcription factor of OCN^38–40^, we investigated the effect of HIF-2α on RUNX2 activity. For a direct readout of RUNX2 activity, we used two types of RUNX2-responsive luciferase reporters (6xOSE-luc and OG2-luc)^41,42^. The increase in RUNX2 activity observed during AA/β-Gp-mediated osteoblast differentiation was dose-dependently inhibited by Ad-*Hif-2α*-mediated HIF-2α overexpression (Fig. 2f). We hypothesized that specific target genes of HIF-2α might inhibit RUNX2-mediated *Ocn* expression during osteoblast differentiation. Based on a previous report that TWIST directly regulates RUNX2 expression and controls the osteogenic differentiation of human mesenchymal stem cells^43^, we examined the transcript levels of *Twist* isotypes in HIF-2α-overexpressing cells. Among the *Twist* isotypes, we found that *Twist2* expression was notably increased by HIF-2α overexpression (Fig. 2g). To verify the in vivo association between HIF-2α and TWIST2, immunohistochemical analysis was applied to examine TWIST2 expression in *Hif-2α*^+/−^ mice (Fig. 2h). Moreover, siRNA-mediated silencing of *Twist2* blocked the HIF-2α overexpression-mediated downregulation of *Ocn* and *Runx2* (Fig. 2i). In an attempt to verify whether *Twist2* is a specific target of HIF-2α, we identified two putative HIF-2α binding sites [5′-(A/G)CGTG-3′] within the promoter region of *Twist2*. Chromatin immunoprecipitation (ChIP) assays performed using two primer pairs designed to span the putative binding sites revealed that HIF-2α directly binds to the promoter region of *Twist2* (Fig. 2j). Strong binding was observed when HIF-2α was overexpressed, while a faintly visible band was detected in control cells during in vitro osteogenic differentiation. In addition, the effects of *Twist2* suppression via siRNA-mediated silencing were evaluated in HIF-2α-induced blockade of bone regeneration. As shown in Fig. 2k, *Twist2* knockdown significantly inhibited the actions of HIF-2α in osteoblasts in vivo. Indeed, the increase in BMP-2-stimulated bone regeneration was synergistically enhanced by *Twist2* knockdown in *Hif-2α*^+/−^ calvaria compared with the regeneration in the WT mice (Supplementary Fig. 4). These data indicate that HIF-2α is upregulated during osteoblast differentiation and that it inhibits osteoblast differentiation by increasing TWIST2 expression to downregulate OCN and RUNX2.

### HIF-2α increases osteoblast-mediated osteoclastogenesis

**Fig. 3:**
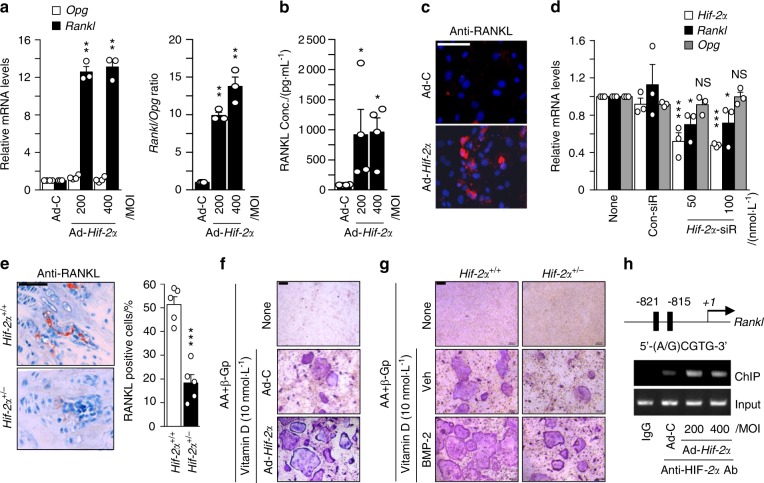
HIF-2α increases osteoblast-mediated osteoclastogenesis. **a**–**c** Primary calvarial preosteoblasts were infected with Ad-C or Ad-*Hif-2α*. qRT-PCR analysis of *Rankl*, *Opg* (left panel), and the *Rankl*/*Opg* ratio (right panel) (*n* = 3; **a**). ELISA analysis of secreted RANKL in culture media (*n* = 4; **b**) and detection of RANKL by immunofluorescence microscopy (scale bar: 100 μm; **c**). **d** Determination of *Hif-2α, Rankl*, and *Opg* expression following siRNA-mediated knockdown of HIF-2α in L-AA/β-GP-induced osteoblasts (*n* = 3). **e** RANKL immunostaining in bone obtained from *Hif-2α*^+/+^ and *Hif-2α*^+/−^ mice. Scale bar: 100 μm. Rankl-positive osteoblasts were counted in the epiphyseal bone compartment. **f** Primary calvarial preosteoblasts infected with Ad-*Hif-2α* and BMMs were mixed and cocultured on glass covers, and TRAP staining was performed. **g** Osteoclasts derived from WT mice and osteoblasts from *HIF-2*α^+/+^ or *HIF-2*α^+/−^ mice in the absence or presence of BMP-2 (100 ng/ml) were cocultured, and TRAP staining was performed (scale bar: 100 μm). **h** A ChIP assay of Ad-*Hif-2α*-infected osteoblasts was performed with anti-HIF-2α antibody and a primer pair designed to span the putative HIF-2α binding regions within the promoter of *Rankl*. Values are presented as the mean ± SEM (*P *<* 0.05, **P *<* 0.01, and ***P *<* 0.005; NS not significant)

As an alternative means to explain the reverse function of HIF-2α, even though its expression was increased during osteoblast differentiation, we paid attention to the upsurge in *Rankl* expression between days 6 and 15 of in vitro culture, which was similar to the upsurge in *Hif-2α* expression (Fig. 2a). RANKL critically enables osteoblasts to regulate osteoclast development and thereby maintain bone mass^44^. Thus, we hypothesized that HIF-2α modulates RANKL expression and RANKL-mediated osteoclastogenesis. We found that HIF-2α overexpression did not affect *Opg* (a decoy receptor for RANKL) but enhanced the transcript level of *Rankl* and the ratio of *Rankl* to *Opg* during the differentiation of calvarial preosteoblasts (Fig. 3a). The protein level of RANKL was increased on day 6 of osteoblastic differentiation, as determined by ELISA of the culture media (Fig. 3b) and immunofluorescence staining (Fig. 3c). siRNA-mediated silencing of HIF-2α in primary cultured calvarial osteoblasts confirmed that HIF-2α regulates *Rankl* expression but not *Opg* expression (Fig. 3d). The immunohistochemistry results consistently showed that RANKL is downregulated upon *Hif-2α* knockdown (Fig. 3e). To examine the ability of HIF-2α to induce osteoclast differentiation by mature osteoblasts and thus contribute to maintaining bone mass^44^, we cultured calvarial preosteoblasts with bone marrow-derived macrophages (BMMs) in the presence of Vitamin D. When cells were cocultured on the same glass covers, we observed more positive TRAP staining among BMMs and Ad-*Hif-2α*-infected preosteoblast cocultures than in cocultures performed with control virus-infected preosteoblasts (Fig. 3f). We also performed cocultures of osteoclasts derived from WT mice and osteoblasts from *Hif-2α*^+/+^ or *Hif-2α*^+/−^ mice. Osteoblast-mediated osteoclastogenesis was dramatically alleviated in *Hif-2α*-deficient osteoblasts relative to WT cells (Fig. 3g).

We further investigated whether RANKL is a specific target of HIF-2α. A ChIP assay using primer pairs designed to span the putative binding sites (−821~−815) within the RANKL promoter revealed that HIF-2α directly binds the RANKL promoter region (Fig. 3h). Taken together, these data suggest that the upregulation of HIF-2α in preosteoblasts increases the osteoclast differentiation of BMMs by increasing RANKL expression.

### Osteoblast-specific depletion of HIF-2α increases bone mass by affecting both osteoblasts and osteoclasts

**Fig. 4:**
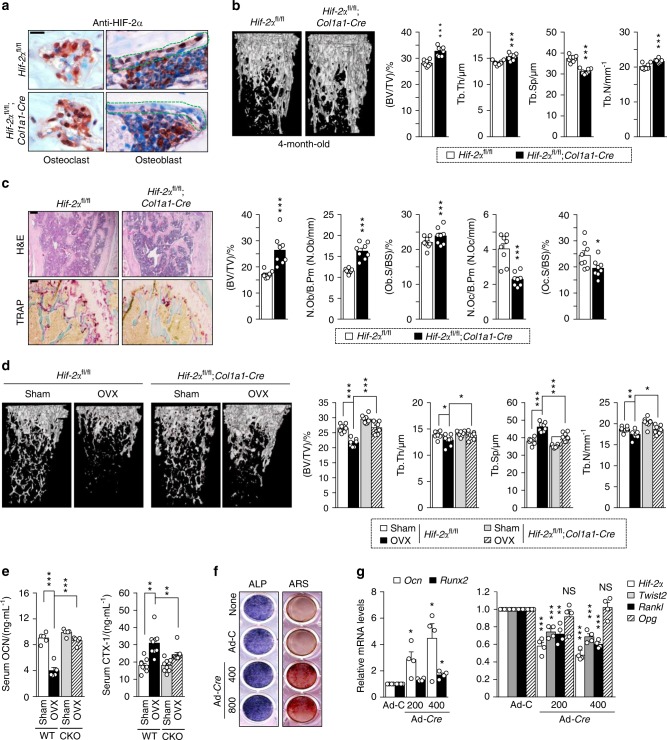
Osteoblast-specific depletion of HIF-2α increases bone mass. **a** Osteoblast-specific depletion of HIF-2α in *Hif-2α*^fl/fl^ and *Hif-2α*^fl/fl^;*Col1a1*-*Cre* mice was determined by immunohistochemistry with anti-HIF-2α antibody. Scale bar: 10 μm **b**, **c** Analyses of femoral trabecular bones from 4-month-old *Hif-2α*^fl/fl^ and *Hif-2α*^fl/fl^;*Col1a1*-*Cre* mice. Representative images of µCT reconstructions of trabecular bones (**b**) and H&E and TRAP staining (**c**). BV/TV, Tb.Th, Tb.Sp, and Tb.N were assessed based on the µCT measurements (*n* = 8; **b**), and BV/TV, N.Ob/B.Pm, Ob.S/BS, N.Oc/B.Pm, and Oc.S/BS were determined from the bone histomorphometric analysis of the metaphyseal regions of femurs (*n* = 8; **c**). Scale bar: 100 μm. **d**, **e** Quantitative µCT analysis of femoral trabecular bones (*n* = 8; **d**) and ELISA-based measurement of the serum concentrations of OCN (*n* = 5; **e**) and CTX-1 (*n* = 9; **e)** in OVX- or sham-operated 3-month-old *Hif-2α*^fl/fl^ and *Hif-2α*^fl/fl^;*Col1a1*-*Cre* mice. **f**, **g** Osteoblast differentiation was validated in primary calvarial preosteoblasts from *Hif-2α*^fl/fl^ mice infected with Ad-C or Ad-*Cre* in the presence of differentiation medium. Osteoblast differentiation was examined by ALP and ARS staining (**f**), and its corresponding gene expression was determined by qRT-PCR (*n* = 4; **g**). Values are presented as the mean ± SEM (*P < 0.05; **P < 0.01, and ***P < 0.005). The effects of OVX and osteoblast-specific deletion of *Hif-2α* (cΚΟ) as well as their interaction in mice were analyzed by two-way ANOVA (**d** BV/TV: interaction = 0.034 1, OVX < 0.000 1, cΚΟ < 0.000 1; **e** OCN: interaction < 0.000 1, OVX < 0.000 1, cΚΟ < 0.000 1; **e** CTX-1: interaction = 0.041 4, OVX < 0.000 1, cΚΟ = 0.046 1)

To further investigate the dual effects of osteoblast-derived HIF-2α on osteoblast differentiation and osteoclastogenesis in an in vivo system, we crossed *Hif-2α*^fl/fl^ mice with *Col1a1*-*Cre* transgenic mice to obtain osteoblast-specific HIF-2α-deficient mice. Immunohistochemical staining with anti-HIF-2α and anti-OCN antibodies confirmed the osteoblast-specific depletion of HIF-2α in *Hif-2α*^fl/fl^;*Col1a1*-*Cre* mice and showed that these mice maintained HIF-2α expression in their osteoclasts (Fig. 4a and Supplementary Fig. 5a). Consistent with our findings in *Hif-2α*^+/−^ mice (Fig. 1), the μCT images and quantitative results (BV/TV, Tb.Th, Tb.Sp, and Tb.N) indicated that the bone mass and trabecular bone percentages were higher in the 4-month-old *Hif-2α*^fl/fl^;*Col1a1*-*Cre* mice than in the age-matched *Hif-2α*^fl/fl^ mice (Fig. 4b). H&E-stained images and bone histomorphometric analyses also showed that the trabecular percentage was increased in the *Hif-2α*^fl/fl^;*Col1a1*-*Cre* mice (Fig. 4c). Notably, TRAP staining and the values of the relevant quantitative parameters, such as N.Oc/B.Pm and Oc.S/BS, showed that bone resorption was decreased in osteoblast-specific *Hif-2α*-null mice (Fig. 4c). Thus, the depletion of HIF-2α from osteoblasts affected the number of osteoclasts. These phenomena were confirmed in OVX model mice. OVX-induced bone resorption was reduced in *Hif-2α*^fl/fl^;*Col1a1*-*Cre* mice versus that in *Hif-2α*^fl/fl^ littermates, as determined by μCT analysis (Fig. 4d), H&E staining, TRAP staining, and bone histomorphometric analyses (Supplementary Fig. 5b). Moreover, the serum OCN level was significantly higher in the *Hif-2α*^fl/fl^;*Col1a1*-*Cre* mice, whereas serum CTX-1 was lower in osteoblast-specific HIF-2α-null mice than in the control littermates. (Fig. 4e). However, *Vegf* expression (Supplementary Fig. 5c) and the number of CD31-positive blood vessels (Supplementary Fig. 5d) were not altered in osteoblast-specific conditional KO bone, suggesting that HIF-2α in osteoblasts is not involved in the angiogenesis mediated by osteoblast-derived VEGF expression. Consistent with the results shown in Figs. 2 and 3, osteoblast differentiation (Fig. 4f) was enhanced by Ad-*Cre* infection in osteoblasts obtained from *Hif-2α*^fl/fl^ mice. Upregulation of *Ocn* and *Runx2* and downregulation of *Twist2* and *Rankl* were noted in the osteoblast-specific HIF-2α KO conditions (Fig. 4g).

### HIF-2α expression in osteoclasts promotes osteoclast differentiation and maturation

**Fig. 5:**
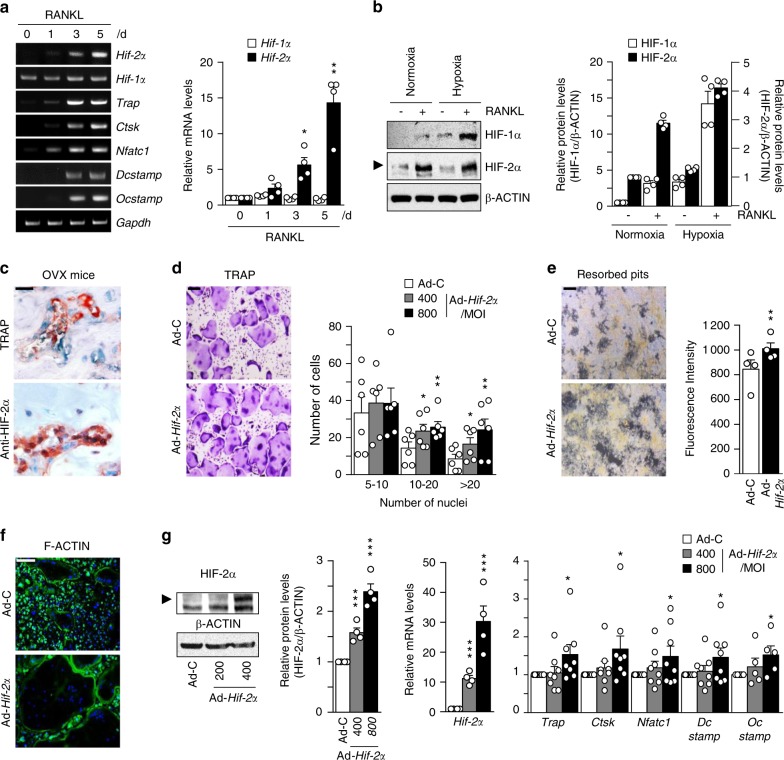
HIF-2α upregulation stimulates osteoclast differentiation. **a** The mRNA levels of osteoclast-related genes during the M-CSF/RANKL-induced osteoclastogenesis of BMMs (*n* = 4). **b** Protein levels of HIF-1α and HIF-2α in BMMs cultured with or without 100 ng·mL^−1^ RANKL for 4 days under normoxia or hypoxia were examined by western blot analysis (left panel) and quantified by ImageJ (right panel) (*n* = 4). **c** TRAP staining and immunohistochemical staining of HIF-2α in serial paraffin sections of femoral trabecular bones from OVX-operated mice. Scale bar: 10 μm **d**–**g** BMMs were infected with Ad-C or Ad-*Hif-2α* and then cultured with M-CSF and RANKL for 5 days. TRAP staining and quantitative analysis of multinucleated cells are shown (*n* = 6) (**d**); mineral resorption pits in differentiated osteoclasts, as quantified by fluorescence intensity (*n* = 4) (**e**); F-actin ring formation (*n* = 3; **f**); and western blotting of HIF-2α and qRT-PCR analysis of *Hif-2α* and the osteoclast-related genes *Trap*, *Ctsk*, *Nfatc1*, *Dcstamp*, and *Ocstamp* (*n* ≥ 4; **g**). Scale bar: 100 μm. Values are presented as the mean ± SEM (*P < 0.05, **P < 0.01, and ***P < 0.005)

**Fig. 6:**
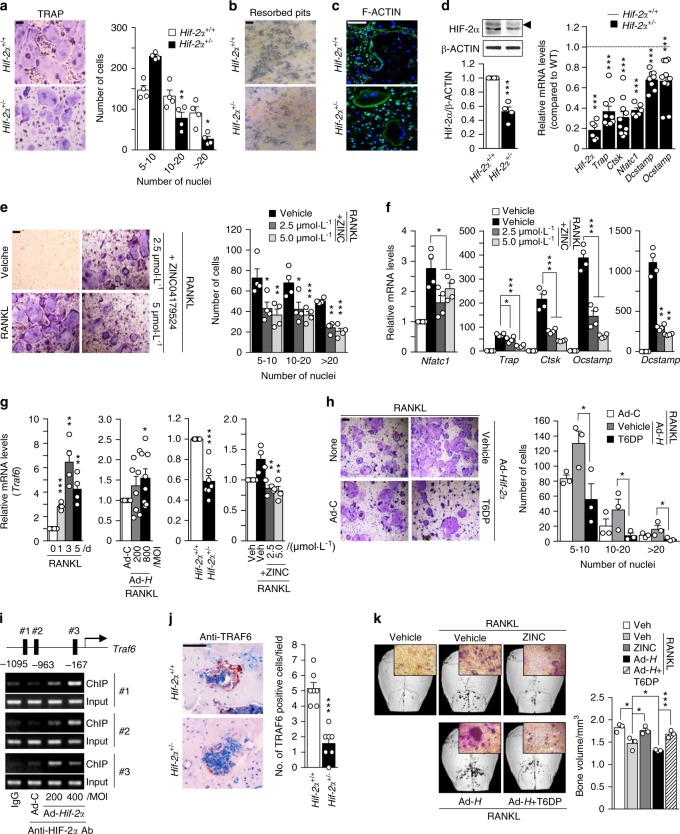
HIF-2α promotes osteoclast function by upregulating TRAF6 expression. **a**–**d** BMMs isolated from 8-week-old *Hif-2α*^+/+^ and *Hif-2α*^+/−^ mice were subjected to M-CSF/RANKL-induced differentiation in vitro. TRAP staining and quantitative analysis of multinucleated cells are shown (*n* = 4; **a**); mineral resorption pits (*n* = 4; **b**); F-actin ring formation (*n* = 4; **c**); and western blotting of HIF-2α / qRT-PCR analysis of *Hif-2α* and osteoclast-related genes (*Trap*, *Ctsk*, *Nfatc1, Dcstamp*, and *Ocstamp*) (*n* ≥ 4; **d**). **e**, **f** TRAP staining and quantification of multinucleated cells were performed in BMMs treated with 2.5 or 5 μmol·L^−1^ ZINC04179524, a potent inhibitor of HIF-2α, during RANKL-mediated osteoclast differentiation (*n* = 4; **e**), and the RNA expression levels of osteoclast-related genes were analyzed by qRT-PCR (*n* = 4; **f**). **g** *Traf6* expression was determined by qRT-PCR during the RANKL-mediated osteoclastogenesis of BMMs in Ad-*Hif-2α*-infected BMMs isolated from WT mice, in BMMs isolated from *Hif-2α*^+/−^ mice, and in BMMs treated with ZINC04179524 (*n* = 4). **h** BMMs infected with Ad-C or Ad-*Hif-2α* with or without 30 μmol·L^−1^ T6DP were analyzed by TRAP staining and quantification of multinucleated cell numbers (*n* = 3). **i** A ChIP assay of Ad-*Hif-2α*-infected osteoclasts was performed with anti-HIF-2α antibody and a primer pair designed to span the putative HIF-2α binding regions within the promoter of *Traf6*. **j** TRAF6 immunostaining in osteoclasts from the bone tissue of *Hif-2α*^*+/−*^ and WT mice. **k** Mice calvaria were injected with RANKL (5 µg) and coinjected with 100 μmol·L^−1^ ZINC04179524 or 100 μmol·L^−1^ T6DP in the presence of Ad-*Hif-2α* (1 × 10^9^ CFU). Calvaria bone resorption was detected by μCT analysis and TRAP staining and quantified by bone volume measurement. Values are presented as the mean ± SEM (*P < 0.05, **P < 0.01, ***P < 0.005). Scale bar: 100 μm

Next, we investigated the expression of HIF-2α during the osteoclast differentiation of BMMs. We used M-CSF and RANKL to induce the osteoclastogenesis of BMMs and monitored the increase in the osteoclast-related marker genes *Trap*, *Ctsk*, *Nfatc1*, *Dcstamp*, and *Ocstamp*. *Hif-2α* expression increased significantly on days 3–5 of in vitro differentiation culture, whereas *Hif-1α* expression was constantly low throughout the culture period (Fig. 5a). The protein level of HIF-1α in RANKL-treated osteoclasts was markedly increased under hypoxia but not normoxia, whereas HIF-2α was upregulated in RANKL-treated osteoclasts under both normoxia and hypoxia (Fig. 5b). Strong HIF-2α expression in osteoclasts was also observed during OVX-induced bone resorption, as assessed by anti-HIF-2α and TRAP immunostaining (Fig. 5c). We next assessed whether HIF-2α directly contributed to osteoclast-mediated bone resorption. We found that HIF-2α alone was not able to induce the osteoclastogenesis of BMMs (data not shown). Therefore, we examined the effects of HIF-2α overexpression in the presence of M-CSF and RANKL. BMM cells were infected with Ad-*Hif-2α* for 24 h and further cultured with M-CSF and RANKL for 5 days. HIF-2α overexpression significantly enhanced osteoclast differentiation, as evidenced by an increase in the number of TRAP-positive multinucleated cells (Fig. 5d). Notably, HIF-2α overexpression yielded very large osteoclasts with a very large cytoplasmic compartment, indicating that HIF-2α stimulates osteoclast maturation (Fig. 5d). Resorption pit analyses showed that HIF-2α overexpression was associated with a large area of mineral resorption (Fig. 5e), and actin ring formation was markedly increased in HIF-2α-overexpressing BMMs (Fig. 5f). These findings suggest that HIF-2α modulates bone resorption by promoting the osteoclast differentiation of BMMs. Consistent with this notion, HIF-2α expression enhanced the expression levels of osteoclast-marker genes, such as *Trap*, *Ctsk*, and *Nfatc1*, and osteoclast-fusion-related genes, such as *Dcstamp* and *Ocstamp*, during the M-CSF/RANKL-induced osteoclastogenesis of BMMs (Fig. 5g). To further confirm the roles of HIF-2α in osteoclast differentiation and function, we examined the osteoclast differentiation and maturation of primary cultured BMMs isolated from *Hif-2α*^+/−^ mice and WT littermates. The TRAP-positive multinucleated cells (Fig. 6a), mineral resorption (Fig. 6b), actin ring formation (Fig. 6c), and osteoclast-related marker gene expression (Fig. 6d) were all lower in cells from *Hif-2α*^+/−^ mice than in those from *Hif-2α*^+/+^ littermates. In addition, we examined how the specific inhibition of HIF-2α with ZINC04179524 (CHEMBL2311967, https://www.ebi.ac.uk/chembl/target/results/keyword) affected osteoclast differentiation. The inhibitory potency of ZINC04179524 was verified by the use of a HIF-2α-responsive luciferase reporter (4xHRE-luc) in IL-1β-treated chondrocytes (Supplementary Fig. 6a) or Ad-*Hif-2α*-infected chondrocytes (Supplementary Fig. 6b). Inhibition of HIF-2α with ZINC04179524 significantly blocked the RANKL-mediated osteoclast differentiation of BMMs, as shown by TRAP staining (Fig. 6e). The number of nuclei in osteoclasts (Fig. 6e) and osteoclast-related gene expression (Fig. 6f) were also dose-dependently reduced by the inhibition of HIF-2α. We further elucidated the underlying molecular mechanism by examining tumor necrosis factor receptor-associated factor 6 (encoded by *Traf6*). TRAF6 is a pivotal component of the RANK signaling pathway, and *Traf6*-deficient mice exhibit severe osteopetrosis, defects in bone remodeling and tooth eruption caused by impaired osteoclast function^45,46^. Indeed, we found that *Traf6* expression was upregulated during RANKL-mediated osteoclast differentiation, and this increased expression of *Traf6* was enhanced by HIF-2α overexpression (Fig. 6g). Reduced expression of *Traf6* was observed in osteoclasts derived from *Hif-2α*^+/−^ mice and HIF-2α inhibitor-treated BMMs (Fig. 6g). Moreover, inhibition of TRAF6 with a TRAF6 decoy peptide (T6DP) significantly blocked the HIF-2α-mediated enhancement of osteoclast differentiation and maturation, as evidenced by a decrease in TRAP-positive multinucleated cells (Fig. 6h). A ChIP assay using three primer pairs designed to span the putative binding sites revealed that HIF-2α directly regulates the expression of *Traf6* (Fig. 6i). To verify the in vivo association between HIF-2α and TRAF6, we initially examined the downregulation of TRAF6 expression in *Hif-2α*^+/−^ mice using immunohistochemistry (Fig. 6j). Next, we evaluated whether HIF-2α inhibition is sufficient for RANKL-induced osteoclast activation and whether TRAF6 is essential for HIF-2α-mediated osteoclastogenesis in vivo. To this end, we examined the in vivo activity of HIF-2α and TRAF6 inhibitors on osteoclast activation using a RANKL-injection-induced bone resorption model in mouse calvaria. Administration of a HIF-2α inhibitor blocked RANKL-induced osteoclastogenesis, which was observed as a decrease in TRAP-positive osteoclasts. HIF-2α overexpression via Ad-*Hif-2α* triggered significant bone resorption, whereas TRAF6 inhibition in vivo with T6DP blocked the HIF-2α-mediated stimulation of osteoclastogenesis (Fig. 6k). Together, our results show that HIF-2α is upregulated in osteoclasts and osteoblasts and promotes osteoclast differentiation/maturation and progressive bone loss.

### Osteoclast-specific depletion of HIF-2α increases bone mass by affecting osteoclasts but not osteoblasts

**Fig. 7:**
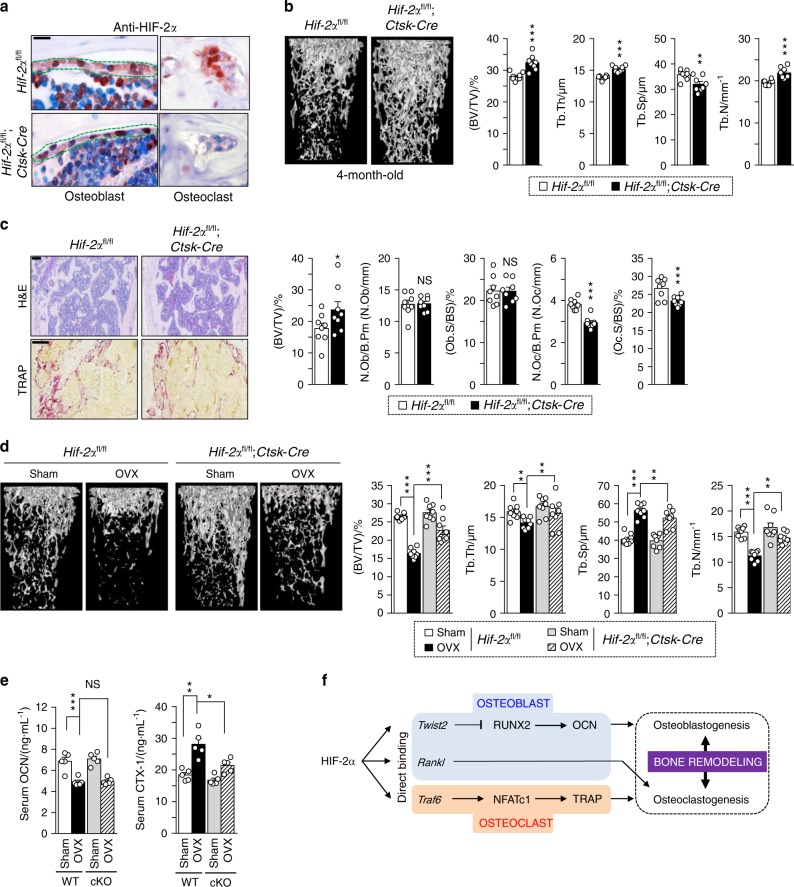
Osteoclast-specific depletion of HIF-2α increases bone mass. **a** Osteoclast-specific depletion of HIF-2α in *Hif-2α*^fl/fl^ and *Hif-2α*^fl/fl^;*Ctsk*-*Cre* mice was determined by immunohistochemistry with anti-HIF-2α antibody (*n* = 3; scale bar: 10 μm). **b**, **c** Analysis of femoral trabecular bones from 4-month-old *Hif-2α*^fl/fl^ and *Hif-2α*^fl/fl^;*Ctsk-Cre* mice. Quantitative µCT analysis of trabecular bones (*n* = 8; **b**), H&E and TRAP staining and bone histomorphometric analysis (*n* = 8; **c**). Scale bar: 100 μm. **d**, **e** Quantitative µCT analysis (*n* = 8; **d**) and measurement of serum OCN and CTX-1 concentrations (*n* = 6; **e**) in OVX or sham-operated *Hif-2α*^fl/fl^ and *Hif-2α*^fl/fl^;*Ctsk-Cre* mice. **f** Schematic diagram depicting HIF-2α regulation of bone remodeling. Values are presented as the mean ± SEM (*P < 0.05, **P < 0.01, ***P < 0.005; ‘NS’ not significant). Scale bar: 100 μm. The effects of OVX and osteoclast-specific depletion of *Hif-2α* (cKO) as well as their interaction in mice were analyzed by two-way ANOVA (**d** BV/TV: interaction = 0.001 8, OVX < 0.000 1, cKO < 0.000 1; **e** OCN: interaction = 0.743 6, OVX < 0.000 1, cKO = 0.557 7; **e** CTX-1: interaction = 0.045 0, OVX < 0.000 1, cKO = 0.002 5)

To further confirm the effects of osteoclast-derived HIF-2α in vivo, we generated osteoclast-specific HIF-2α-deficient mice by crossing *Hif-2α*^fl/fl^ mice with *Ctsk*-*Cre* mice. Immunostaining confirmed that the osteoblasts of these mice stained positive for HIF-2α, whereas the osteoclasts did not (Fig. 7a). Indeed, double immunostaining of HIF-2α and CTSK revealed nuclear localization of HIF-2α in osteoclasts (Supplementary Fig. 7a). μCT and quantitative analyses revealed that this osteoclast-specific depletion of HIF-2α increased the BV/TV (Fig. 7b). Bone histomorphometric analyses with H&E and TRAP staining showed that N.Oc/B.Pm and Oc.S/BS were significantly decreased in the *Hif-2α*^fl/fl^;*Ctsk*-*Cre* mice, whereas N.Ob/B.Pm and Ob.S/BS were unaltered (Fig. 7c). This finding indicates that the specific depletion of HIF-2α in osteoclasts increased the relative bone volume by preventing bone resorption. We further found that OVX-induced bone resorption (Fig. 7d and Supplementary Fig. 7b) and the levels of serum CTX-1, but not serum OCN, were significantly lower in OVX *Hif-2α*^fl/fl^;*Ctsk*-*Cre* mice than in OVX *Hif-2α*^fl/fl^ mice (Fig. 7e). From these data, we conclude that the expression of HIF-2α in osteoclasts promotes osteoclast activation in addition to its role in osteoblasts (Fig. 7f).

## Discussion

Pathological bone diseases are caused by dysregulation of the interplay between osteoblasts and osteoclasts, as well as by an imbalance between osteoclast-mediated bone resorption and osteoblast-mediated bone formation. In the current study, we show that HIF-2α deficiency increased bone mass by promoting osteoblast differentiation and inhibiting osteoclast differentiation. Moreover, coculture experiments and analyses of *Hif-2α-*conditional KO mice showed that HIF-2α-mediated RANKL expression in osteoblasts affected the differentiation and maturation of osteoclasts. The RANKL secreted by preosteoblasts infected with Ad-*Hif-2α* was sufficient to induce the differentiation of BMMs. The osteoblast-specific depletion of HIF-2α in *Hif-2α*^fl/fl^;*Col1a1*-*Cre* mice increased bone mass by affecting both osteoblasts and osteoclasts, whereas the osteoclast-specific loss of HIF-2α in *Hif-2α*^fl/fl^;*Ctsk*-*Cre* mice increased bone mass by affecting osteoclasts but not osteoblasts. Thus, HIF-2α appears to critically regulate the interplay between osteoblasts and osteoclasts by directly increasing RANKL expression in preosteoblasts.

On the cellular level, HIF-2α expression increased on day 3 of the in vitro osteogenic differentiation of mouse calvarial osteoblast precursor cells, remained steady until day 15, and decreased thereafter. The expression levels of *Ocn*, which has been implicated in bone mineralization, and *Runx2*, which is a key transcription factor associated with osteoblast differentiation, showed the reverse pattern, undergoing upregulation between days 18 and 24 when HIF-2α was downregulated. Based on this observation, we hypothesized that a target gene of HIF-2α reduces the expression levels of RUNX2 and OCN. Several lines of evidence suggest that TWIST negatively regulates RUNX2 expression and activity, which is followed by the sequential downregulation of OCN expression^43,47^. In particular, hypoxia and HIF-1α inhibit the expression of type 1 RUNX2 via TWIST, which is a downstream target of HIF-1α; this inhibition further inhibits BMP-2 expression, type 2 RUNX2 expression, and osteoblast differentiation in human mesenchymal stem cells^43^. In the present study, we determined that *Twist2* acts as a direct target gene of HIF-2α to inhibit RUNX2 expression, which may decrease OCN expression and inhibit mineralization by osteoblasts, resulting in decreased bone mass. The role of HIF-2α in osteoblast-mediated osteoclastogenesis was additionally examined in the current study. Interestingly, we obtained results opposite to those of Wu et al.^48^, who previously reported that *Opg* is a target gene of HIF-2α but not *Rankl*; the reasons underlying the inconsistent findings between the studies are unclear. Based on our data, we propose that HIF-2α protein in osteoblasts directly binds the promoter of *Rankl* to promote osteoclast differentiation. This proposal is supported by our previous findings of HIF-2α-mediated upregulation of RANKL in fibroblast-like synoviocytes and abrogation of RANKL-induced bone resorption in the region of RA pannus in heterozygous *Hif-2α* KO mice ^32^.

Numerous hormones, cytokines, and growth factors play pivotal roles in osteoclast development. Based on our present results, we suggest that HIF-2α is a previously unrecognized catabolic factor of osteoclast differentiation and maturation. To address the molecular mechanism underlying HIF-2α-stimulated osteoclastogenesis, we used adenoviral infection to overexpress HIF-2α and analyzed molecular markers of osteoclast differentiation and cell fusion. We found that Ad-*Hif-2α* infection upregulated various genes involved in both osteoclast differentiation (e.g., *Trap*, *Ctsk*, and *Nfatc1*) and osteoclast fusion (e.g., *Dcstamp* and *Ocstamp*)^49^. Here, we suggest that TRAF6 acts as a key connecting protein in HIF-2α-induced gene expression because it acts as the crucial adaptor molecule of RANK, leads to the induction of *Nfatc1*, and is critical for osteoclastogenesis^50^. This idea is supported by a previous report that *Traf6* is a direct target gene of HIF^51^ and by the results of our experiments using cell-permeable T6DP in the presence of Ad-*Hif-2α* (Fig. 6h, k).

Given the hypoxic nature of the bone microenvironment, it has been suggested that hypoxia and HIF play critical roles in bone formation. Of the three α subunits of HIF (HIF-1α, HIF-2α, and HIF-3α), HIF-1α has been intensively studied both in the normal physiology of bone homeostasis and in pathological bone diseases. The α subunit of the HIF proteins is hydroxylated on an oxygen-dependent degradation domain by oxygen-sensing PHD enzymes under normoxia; thereafter, the binding of E3 ligase von Hippel–Lindau protein (pVHL) to hydroxylated HIF-α subunits is followed by their polyubiquitination and proteasomal degradation^52^. Wang and coworkers demonstrated that activation of the HIF-α pathway in osteoblasts of *Vhl*-KO (Δ*Vhl*) mice produces high levels of *Vegf*, leading to the development of dense and heavily vascularized long bones^22^. In addition, Shomento et al.^24^ observed decreased bone volume in mice lacking HIF-1α in osteoblasts, suggesting that HIF-1α is critical for coupling angiogenesis to osteogenesis during endochondral ossification. However, the potential role of HIF-1α in the regulation of osteoblast–osteoclast crosstalk during osteoporotic bone loss is controversial. Mice with genetic ablation of *Phd* in osteoblasts showed crosstalk between osteoblasts and osteoclasts via overexpression of OPG, an HIF target gene that regulates bone homeostasis and protects against OVX-induced bone loss^48^. This finding was supported by the observation that specific disruption of VHL in osteoblasts and the subsequent activation of HIF signaling in these cells could protect against OVX-induced bone loss. In osteoclastogenesis, in contrast, HIF is reportedly required for osteoclast formation and bone resorption^53^, and osteoclast-specific HIF-1α depletion in mice was shown to antagonize OVX-induced (in female) or ORX-induced (in male) bone loss ^26,27^.

Distinct functions of HIF-2α and HIF-1α in bone development and osteoblast functions have been proposed^22,24,48^. Specific deletion of either HIF-1α or HIF-2α in osteoblasts led to a similar increase in VEGF-mediated skeletal vascularity, whereas HIF-1α, but not HIF-2α, enhanced bone formation by regulating osteoblast differentiation and proliferation^24^. In addition, only HIF-1α exerted tumorigenic effects on bone tissues, and genetic invalidation of HIF-2α in osteoblast-lineage cells did not significantly modulate the bone phenotype in young mice^22^. Results distinct from those of other reports were obtained in the current study. Heterozygous *Hif-2α* KO and osteoblast-specific *Hif-2α* depletion in mice led to a significant increase in bone mass through modulation of both osteoblasts and osteoclasts. This discrepancy may be explained in two ways. First, we used a different *Cre* transgenic model, *Col1a1*-*Cre*, to generate osteoblast-specific conditional KO mice, rather than the *Ocn*-*Cre*^22,24^ or *Osx*-*Cre*^48^ transgenic mice used by the other groups. *Col1a1* is expressed earlier than *Ocn* during osteogenesis^54^. To further elucidate the expression patterns of osteogenic markers and HIF-2α, we examined *HIF-2α*, *COL1A1*, and *OCN* levels during osteogenesis in human mesenchymal stem cells (data not shown). Consistent with the results obtained using preosteoblast cells (Fig. 2a), the mRNA expression pattern of *Hif-2α* was very similar to that of *Col1a1* but decreased at the *Ocn*-expression stage. In addition, it has been reported that different maturation stages of osteoblasts can be targeted by *Osx*-*Cre* and 2.3 kb *Col1a1*-*Cre* transgenic mice. This information supports the different phenotypes observed between our study and earlier studies. Another possible explanation for the inconsistent results is the differences in the ages of mice used for the experiments. While the other groups analyzed juvenile or young adult (6 to 8 weeks old) mice, we used mature mice to evaluate the regulatory role of HIF-2α in bone remodeling and osteoporotic bone loss. VEGF-mediated control of angiogenesis–osteogenesis coupling is a major critical factor in bone development. To elucidate this issue, we additionally analyzed bone volumes in younger mice (4 and 8 weeks old). Interestingly, no significant changes in bone mass were evident, suggesting that HIF-2α functions as a pivotal molecule in bone remodeling and not bone modeling (Supplementary Fig. 2). In the current study, HIF-2α induced only a modest increase in *Vegf* expression in osteoblasts compared to that induced by HIF-1α (data not shown). However, the potential angiogenesis-independent roles of HIF-2α in bone remodeling remain to be elucidated. Given the collective findings, we hypothesize that HIF-1α plays a predominant role in regulating angiogenesis–osteogenesis coupling under normal physiological conditions, while HIF-2α may contribute to bone remodeling, in part, by regulating interactions between osteoblasts and osteoclasts through modulation of their pivotal markers.

Several reports support distinct functions of HIF-1α and HIF-2α in selected tissues, even though they are homologous and share a conserved oxygen-dependent degradation domain^55–58^. Although the two α subunits are structurally similar and recognize the same DNA element, the target genes regulated by HIF-1α and HIF-2α are not identical^56–58^. A recent report showed that HIF-2α is essential for the endochondral ossification of cultured chondrocytes and embryonic skeletal growth independent of oxygen-dependent hydroxylation^59^. Furthermore, it has been demonstrated that HIF-2α is an essential catabolic regulator of osteoarthritis cartilage destruction and RA pathogenesis^32,60–62^. In the pathogenesis of osteoarthritis, HIF-2α regulates subchondral bone sclerosis, the formation of osteophytes in joints, and RA; this process occurs via the direct or indirect (via IL-6 signaling) upregulation of matrix-degrading catabolic enzymes, such as MMP-3 and MMP-13, and the Fas-mediated apoptosis of chondrocytes^60–62^. HIF-2α also plays key roles in RA pathogenesis by regulating angiogenesis, IL-6-dependent T_H_17 cell differentiation, and fibroblast-like synoviocyte functions^32^. Here, we show that HIF-2α, but not HIF-1α, accumulated during osteoblast differentiation and the RANKL-mediated osteoclastogenesis of BMMs under normoxia (Figs. 2b and 5b). Cytokines and hormones are known to affect the protein accumulation of HIF-1α and may be involved in activating HIF-1α under normoxia^63^. Our previous studies suggested that cytokines (e.g., IL-1β, IL-6, and TNF-α) expressed in articular chondrocytes during the pathogenesis of osteoarthritis and RA increase HIF-2α expression and protein accumulation under normoxia^32,60^. In OVX-induced osteoporosis models, estrogen deficiency increases cytokine expression^64^, which may affect HIF-2α expression and HIF-2α-mediated osteoclast activation.

A number of drugs that inhibit bone resorption are currently used in the clinic. Bisphosphonates, which are the most widely used drugs against osteoporosis, are effective in slowing the progression of osteoporotic bone loss by inhibiting bone resorption. However, there is a limit to the recovery of osteoporosis that has already progressed^65^, and recent reports have demonstrated that rare but serious adverse effects may occur as a result of bisphosphonate therapy^66^. Parathyroid hormone-based drugs, such as teriparatide, are commonly used to treat osteoporosis by promoting osteogenesis. However, these drugs have the disadvantages of being costly and inconvenient to administer, and they may activate bone resorption^67^. To overcome these limitations, researchers have long sought to develop antiosteoporosis drugs that affect bone resorption and prevent the reduction in bone formation. Our present results suggest that HIF-2α is a key regulator in the maintenance of bone homeostasis. The potent HIF-2α inhibitor, ZINC04179524, blocked RANKL-mediated osteoclastogenesis, and osteoclast maturation but failed to block osteoblast differentiation (data not shown). We speculate that HIF-1α and HIF-2α cooperatively contribute to osteoblast differentiation, whereas HIF-2α, but not HIF-1α, has major functions in osteoclast differentiation and activation under normoxia, which can be associated with pathophysiological conditions. In addition, inhibition of TRAF6 with a T6DP completely blocked the effects of HIF-2α overexpression on RANKL-mediated osteoclast differentiation and maturation.

This study revealed an unanticipated molecular mechanism accounting for the regulation of bone remodeling by HIF-2α. In summary, our data suggest that HIF-2α inhibits osteoblastogenesis but drives osteoclastogenesis through direct regulation of TWIST2 or TRAF6. Moreover, HIF-2α appears to act as a critical regulator of the interplay between osteoblasts and osteoclasts by directly increasing RANKL expression (see Fig. 7f). Taken together, our present results suggest that HIF-2α may be a key factor in the maintenance of bone homeostasis, and its regulation may be an important therapeutic target in efforts to address bone fracture and pathological diseases associated with bone loss, including cancer, RA, and osteoporosis.

## Materials and methods

### Mice and experimental models

*Hif-2α*^*+/−*^ and *Hif-2α*^fl/fl^ mice were obtained from Jackson Laboratory (Sacramento, CA, USA), and *Ctsk-C*re mice were obtained from the Rodent Model Resource Center (Taipei, Taiwan). Type I collagen promoter (2.3 kb) (*Col1a1*)*-Cre* mice were kindly provided by Dr Je-Yong Choi (Kyungpook National University, Daegu, Korea)^68^. To generate osteoblast- and osteoclast-specific *Hif-2α-*KO mice, *Hif-2α*^fl/fl^ mice (containing loxP sites flanking exon 2 of *Hif-2α*) were backcrossed against *Col1a1-Cre* and *Ctsk-Cre* mice, respectively. Male mice were used for all experiments except for the OVX models. The OVX models were generated by a 5-mm dorsal incision in 8-week-old female mice; a sham operation was performed as a control. After 4 weeks, the OVX mice were sacrificed for further analysis. For calvarial bone defect models^42^, a critical-sized calvarial defect was created using a 5-mm diameter trephine bur (Fine Science Tools, Foster City, CA, USA) in 6-week-old *Hif-2α*^+/+^, *Hif-2α*^+/−^, and WT C57BL/6 male mice and covered with absorbable collagen sponges containing 300 ng BMP-2 (Cowell Medi Corp., Seoul, Korea). For each purpose, collagen sponges containing Ad-C, Ad-*Hif-2α*, or Ad-sh*Twist2* (1 × 10^9^ CFU) were applied. After 2 weeks, the calvarial model mice were sacrificed for further analysis. Calvarial bone resorption models were created using 8-week-old C57BL/6 male mice^69^. Specifically, collagen sponges soaked with Ad-*C*, Ad-*Hif-2α*, or the indicated inhibitors in the presence of RANKL recombinant protein (5 μg, Peprotech, Rocky Hill, NJ, USA) were implanted into calvarial bone, and mice were sacrificed after 5 days for further analysis. The adenovirus expressing mouse *Hif-2α* (Ad-*Hif-2α*) was described previously^60^. The adenovirus expressing shRNA for silencing mouse *Twist2* was purchased from Vector Biolabs (#shADV-275369; Malvern, PA, USA). All animal experiments were approved by the Institutional Animal Care and Use Committee (IACUC) of Chonnam National University (Gwangju, Korea).

### μCT analysis

μCT images of distal femurs fixed in 10% neutral buffered formalin solution were obtained using a high-resolution Skyscan 1172 system (Bruker, Aartselaar, Belgium). The X-ray source was set to 50 kV and 200 μA, and a 0.5-mm aluminum filter was used. Image reconstruction software (NRecon; Bruker) was used to reconstruct serial cross-section images using identical thresholds for all samples (0–6 000 in Hounsfield units). For the trabecular bone of proximal femurs, we manually designated a region of interest comprising 300 total steps starting 30 steps away from the growth plate. Femoral morphometric parameters were determined with data analysis software (CTAn). Trabecular morphometry was analyzed by measuring the BV/TV, Tb.Th, Tb.N, and Tb.Sp. Three-dimensional surface rendering images were generated using Mimics 14.0 imaging software (Materialise, Plymouth, MI, USA).

### Statistical analysis

All experiments were performed independently at least three times. All quantified data in bar charts with scatter plots are presented as the mean ± SEM. All statistical analyses were performed using GraphPad Prism version 7 software. All quantified data were first tested for conformation to a normal distribution using the Shapiro–Wilk test and were then analyzed by two-tailed Student’s *t*-test (pairwise comparisons) or analysis of variance (ANOVA) followed by Tukey’s post hoc tests (multicomparison), as appropriate. Changes in bone volume and the ELISA results of serum OCN and CTX-1 obtained from OVX mice were analyzed by two-way ANOVA for the effects of genetic deletion and OVX and their interactions. The *n*-value is the number of independent experiments or mice. Significance was accepted at the 0.05 level of probability (*P* < 0.05).

## Supplementary information

Supple information

## Data Availability

The authors declare that the data supporting the findings of this study are available within the article and its supplementary information files, or are available upon reasonable request to the authors.
