# Supplementary material for: Controlling hypoxia-inducible factor-2α is critical for maintaining bone homeostasis in mice
Source: Bone Res. 2019 May 13;7:14. doi: 10.1038/s41413-019-0054-y (PMC6513851; doi:10.1038/s41413-019-0054-y)
Supplement: Supplementary file 1 — Supple information [file 41413_2019_54_MOESM1_ESM.docx]

**Supplementary Information**

**Controlling hypoxia-inducible factor-2α is critical to maintaining bone homeostasis in mice**

Sun Young Lee^1,2†^, Ka Hyon Park^1,2†^, Hyung-Gu Yu^2†^, Eunbyul Kook^2^, Won-Hyun Song^2^, Gyuseok Lee^2^, Jeong-Tae Koh^1,2^, Hong-In Shin^3^, Je-Yong Choi^4^, Yun Hyun Huh^5*^ and Je-Hwang Ryu^1,2*^

Supplementary Materials and Methods

Supplementary Figure 1 to 7

Supplementary Table 1

**Supplementary Materials and Methods**

**Osteogenic differentiation of primary cultured pre-osteoblasts**

For primary culture of calvarial pre-osteoblasts, calvarial bones isolated from 3-day-old pups were enzymatically digested twice with DMEM containing 0.1% type Ⅱ collagenase (Sigma, St. Louis, MO, USA) and 0.25% trypsin/EDTA at 37℃ for 20 min. The isolated cells were cultured in complete DMEM (GIBCO, Grand Island, NY, USA) containing 10% fetal bovine serum (FBS) and 1% penicillin/streptomycin (P/S). After 3 days, osteoblast differentiation was induced by plating 1 x 10^5^ cells per 35-mm dish in osteogenic differentiation media, which consisted of complete media containing 50 μg/ml L-AA (Sigma) and 5 mM β-Gp (Chemcruz, Dallas, TX, USA). Differentiation culture was continued for 24 days with replacement of the medium every other day. For adenovirus-mediated overexpression, adenoviral infection was performed on day 2 by using Ad-C or Ad-*Hif2α* at the indicated 200 or 400 MOI (Multiplicity of infection). For osteoblast-specific deletion of *Hif-2α*, calvarial pre-osteoblast were isolated from *Hif-2α*^fl/fl^ mice and infected with Ad-C or *Cre*-recombinase adenovirus (Ad-*Cre*, #1045, Vector biolabs ) at a MOI of 400 or 800. For siRNA-mediated knockdown, primary calvarial pre-osteoblasts were transfected with mouse *Hif-2α*-siRNA (SMARTpool, #M-040635-01; Dharmacon, La Fayette, CO, USA) or *Twist2*-siRNA (SMARTpool, #M-044881-01; Dharmacon) at the indicated concentrations using Lipofectamine RNAiMAX (Invitrogen, Carlsbad, CA, USA) following the manufacturer's recommended procedures. Non-targeting siRNA (scrambled; #SN-1013; Bioneer, Daejeon, Korea) was used as a negative control.

**Osteoclast differentiation of BMMs**

Bone marrow cells were isolated from the long bones (tibiae and femurs) of 6- to 8-week-old mice by flushing of the bone marrow with serum free α-MEM, and then cultured in complete α-MEM containing 10% FBS and 1% P/S for 24 h. For osteoclast differentiation, non-adherent cells were collected and cultured in complete α-MEM in the presence of 30 ng/ml of M-CSF (Peprotech, Rocky Hill, NJ, USA) for 3 days. Adherent BMMs (4 x 10^4^ cells per well in a 48-well plate) were maintained in complete α-MEM containing 30 ng/ml of M-CSF for 24 h and then replenished with medium containing 100 ng/ml of RANKL (Peprotech) for 5 days. For the inhibition of HIF-2α and TRAF6, ZINC04179524 (VitasMLab, Apeldoorn, Netherlands) and TRAF6 inhibitory peptide (Novus Biologicals, Littleton, CO, USA), respectively, were applied on the first day of RANKL replenishment (designated day 0). Adenoviral infection was performed on day 1. TRAP (Sigma) staining and bone resorption assays (CSR-BRA-48KIT; Cosmo Bio Co., Tokyo, Japan) were performed on day 5 according to the manufacturer’s recommendations. Briefly, cells were fixed and stained using TRAP, and TRAP-positive multinucleated cells (MNCs) were counted as osteoclasts. For the resorption assay, BMMs were seeded on calcium phosphate (CaP)-coated plates. The cells were removed by treating the plates with 5% sodium hypochlorite for 5 min, and the resorption pits were visualized using an inverted phase contrast microscope.

**Co-culture of osteoblasts and osteoclasts**

To induce osteoblast-mediated osteoclast differentiation, primary calvarial pre-osteoblasts isolated from *Hif-2α*^+/+^ or *Hif-2α*^+/-^ infected with Ad-*Hif-2α* were cultured with BMMs isolated from WT mice in the presence of BMP-2 (100 ng/ml), 50 μg/ml L-AA, 5 mM β-Gp and 10 nM 1,25-dihydroxyvitamin D3 for 5 days^1^. Then, cells were fixed and stained for TRAP. Images were captured using a LAS (Leica ApplicationSuite) V4.1 program (Leica, Swizerland).

**Hypoxia induction**

A hypoxic environment containing 2% O_2_ and 5% CO_2_ was induced using a GasPak^TM^ EZ Container System (BD Biosciences, Sparks, MD, USA) according to the manufacturer’s instructions^2^.

**Reverse transcription-polymerase chain reaction (RT-PCR) and quantitative real-time (qRT)-PCR**

Total RNA was prepared using the TRI reagent (Molecular Research, Cincinnati, OH, USA) and reverse transcribed with TOPscript RT DryMIX (Enzynomics, Daejeon, Korea). cDNA was subjected to PCR using the AmpOne^TM^ Tap DNA Polymerase Mix (GeneAll, Seoul, Korea) with appropriate primers (Supplementary Table 1). qPCR was performed using a StepOnePlus Real-Time PCR system (Thermo Fisher Scientific, Waltham, MA, USA) with SYBR Premix Ex Taq (Takara Bio, Kyoto, Japan). For each target gene, the individual transcript levels were normalized to those of *glyceraldehyde-3-phosphate dehydrogenase* (*Gapdh*) and expressed as a fold change relative to the indicated controls.

**Western blotting**

Cells were lysed in lysis buffer containing 50 mM Tris-HCl, pH 8.0, 150 mM NaCl, 5 mM NaF, 1% NP-40, 0.2% SDS, 0.5% deoxycholate, a protease inhibitor cocktail and a phosphatase inhibitor cocktail (Roche, Basel, Switzerland). Proteins were separated by SDS-PAGE and transferred to nitrocellulose membranes. The membranes were blotted with 5% skim milk for 1 h and then incubated with primary antibodies at 4°C overnight. The following antibodies were used: rabbit anti-HIF-2α (NB100-122; Novus Biologicals), mouse anti-HIF-1α (H6536; Sigma), rabbit anti-Lamin B (sc6216, Santa Cruz Biotech., Santa Cruz, CA) and mouse anti-β-Actin (A3584; Sigma). The membranes were incubated with horseradish peroxidase-conjugated anti-mouse or anti-rabbit IgG (Sigma) and detected using ECL solution (GE Healthcare, Little Chalfont, Buckinghamshire, UK). Protein levels were quantified using the NIH ImageJ program (version 1.47, National Institutes of Health, Bethesda, MD).

**Immunohistochemistry and Immunofluorescence microscopy**

Mouse joint tissues were sectioned at 5-μm thickness for immunohistochemical staining. Antigen retrieval was performed by incubating sections with 0.1% trypsin for 40 minutes at 37°C. The following primary antibodies were used for immunohistochemistry: rabbit anti-HIF-2α (NB100-122; Novus Biologicals), mouse anti-TWIST2 (H00117581-M01; Abnova**,** Taipei, Taiwan), and mouse anti-TRAP (NBP2-45293; Novus Biologicals); mouse anti-RANKL (sc52950; Santa Cruz Biotech.); and rabbit anti-TRAF6 (AD1-AAP-426E; Enzo Life Sciences, Farmingdale, NY, USA). For double-immunofluorescence labeling of mouse tissues, the following primary antibodies were used: mouse anti-HIF-2α (sc13596, Santa Cruz Biotech.), rabbit anti-HIF-2α (Novus Biologicals), mouse anti-OCN (OC4-30; Thermo Fisher Scientific), rabbit anti-CTSK (ab19027; Abcam, Cambridge, MA, USA) and rat anti-CD31 (DIA-310; Dianova GmbH, Hambrug, GM). Proteins were visualized using Alexa 488- or Alexa 594-conjugated secondary antibodies (Thermo Fisher Scientific). For immunofluorescence staining, cells grown on 12-mm coverslips were fixed with 3.5% PFA for 10 min and permeabilized with 0.1% Triton X-100 for 10 min. The cells were blocked with 1% BSA for 30 min, incubated for 1 h with primary antibodies and then incubated for 1 h with an Alexa 594-conjugated secondary antibody. F-actin was visualized with Alexa Fluor 488-phalloidin. Anti-tubulin, anti-HIF-2α and anti-RANKL antibodies were used, and nuclei were detected by DAPI (4’,6-diamidino-2-phenylindole). The each image file of slides was analyzed using the NIH ImageJ program to quantify the number of cells stained positively for the specific antibody.

**Enzyme-linked immunosorbent assay (ELISA)**

For quantification of RANKL protein levels in the culture medium, primary calvarial pre-osteoblasts were infected with Ad-C or Ad-*Hif-2α* (200 or 400 MOI). After 24 h, supernatants were collected and the RANKL protein levels were measured using a RANKL-ELISA kit (Abcam) according to the manufacturer’s recommendations. To measure the level of serum OCN and CTX-1, sera were collected from heterozygous *Hif-2α*-KO or osteoblast- or osteoclast-specific *Hif-2α*-cKO mice that had been subjected to OVX. Serum OCN and CTX-1 levels were measured using mouse Gla-OCN High Sensitive EIA kit (MK127; Takara Bio., Shiga, Japan) and RatLaps^TM^ (CTX-1) EIA kit (AC-06F1; IDS, Tyne & Wear, UK) respectively.

**Luciferase reporter assay**

Primary cultured calvarial pre-osteoblasts were infected with Ad-*Hif-2α* and transfected with RUNX2-responsive luciferase reporter vectors, pGL3-6xOSE or pGL3-OG2, plus a cytomegalovirus-β-galactosidase vector as an internal control^3^. After 48 h of transfection, cell lysates were collected and assayed for luciferase activity using a luciferase reporter assay system (Promega, Madison, WI, USA) according to the manufacturer’s protocols. The obtained values were normalized with respect to β-galactosidase activity.

**Chromatin immunoprecipitation (ChIP) assay**

ChIP assays were performed using a Magna ChIP kit (Millipore, Billerica, MA, USA) according to the manufacturer's guidelines. In brief, 1% formaldehyde was added directly to the cell culture medium to crosslink the DNA and proteins, cell lysates were sonicated to fragment the DNA, and the samples were incubated with rabbit anti-mouse HIF-2α antibody (Novus) or control anti-mouse IgG at 4°C overnight. Antibody–protein–DNA complexes were precipitated with protein A–agarose. The utilized PCR primers were designed to amplify the putative HIF-responsive elements within the promoter region of *Twist2*, *Rankl* and *Traf6*, and were shown in Supplementary Table 1.

***In vivo* calcein labeling**

For calcein labeling, mice were intraperitoneally injected with 20 mg/kg calcein (Sigma) at 10 and 3 days prior to sacrifice. Femurs were fixed in 4% paraformaldehyde for 2 days at 4°C, embedded in methyl-methacrylate and sectioned. Images were captured by fluorescence microscopy (Zeiss, Oberkochen, Germany) and analyzed for BFR and MAR by using the Bioquant program (Bio-Quant Inc., San Diego, CA, USA)^4^.

**ALP and ARS staining**

Primary cultured calvarial pre-osteoblasts infected with Ad-C or Ad-*Hif-2α*, and from *Hif-2α*^+/+^ or *Hif-2α*^+/-^mice were maintained in osteogenic differentiation media for 6 days with replacement of the osteogenic differentiation media every 2 day. For the HIF-2α inactivation study on the osteoblast differentiation, primary calvarial pre-osteoblasts isolated from *Hif-2*α^fl/fl^ mice were infected with Ad-C or Ad-*Cre* for 2 h at the indicated MOI. For ALP staining, cells were fixed with 4% formaldehyde, rinsed with deionized water and stained with 5-bromo-4-chloro-3-indolyl phosphate (BCIP®)/nitro blue tetrazolium (NBT) Liquid Substrate solution (Sigma) for 15 min in a dark room. For mineralization assays, cells were fixed with 4% formaldehyde for 1 h and stained with 40 mM Alizarin red S (Sigma) solution for 15 min at room temperature^5^.

**REFERENCES**

1. Wang, L. *et al.* Osteoblast-induced osteoclast apoptosis by fas ligand/FAS pathway is required for maintenance of bone mass. *Cell Death Differ.* **22,** 1654–1664 (2015).

2. Ryu, J. H. *et al.* Hypoxia-Inducible Factor-2α is an essential catabolic regulator of inflammatory rheumatoid arthritis. *PLoS Biol.* **12,** e1001881 (2014).

3. Jeong, B. C. *et al.* The orphan nuclear receptor estrogen receptor-related receptor γ negatively regulates BMP2-induced osteoblast differentiation and bone formation. *J. Biol. Chem.* **284,** 14211–14218 (2009).

4. Kim, J. H. *et al.* Kruppel-like factor 4 attenuates osteoblast formation, function, and cross talk with osteoclasts. *J. Cell. Biol.* **204,** 1063–1074 (2014).

5. Kim, J. W. *et al.* Chemical inhibitors of c-Met receptor tyrosine kinase stimulate osteoblast differentiation and bone regeneration. *Eur. J. Phamacol.* **806,** 10–17 (2017).

**Supplementary Figures**

**Supplementary Fig. 1** Heterozygous *Hif-2α* KO mice show no differences in cortical thickness and only a modest increase in cortical volume. **a, b** Cortical bone volume (BV/TV), area (Ct.Ar), thickness (Ct.Th) and perimeter (Ct.Pm) were assessed from the µCT measurements of cortical bones from 4-month-old mice (n = 8; **a**) and OVX- or sham-operated mice (n = 8; **b**). Values are presented as means ± SEM (* *P*<0.05; ***P*<0.01; ****P*<0.005). ‘NS’, not significant. The effects of OVX and genetic deletion of *Hif-2α* (KO) as well as their interaction in mice were analyzed by two-way ANOVA (**b**, BV/TV: interaction < 0.0001, OVX = 0.2258, KO = 0.0116)

**Supplementary Fig. 2** Bone mass of 4- or 8-week-old *Hif-2α*^+/+^ and *Hif-2α*^+/-^ mice. **a**, **b** Representative images of µCT reconstructions of femoral trabecular bones from 4- (**a**) or 8-week-old (**b**) *Hif-2α*^+/+^ and *Hif-2α*^+/-^ mice. BV/TV was analyzed from the µCT measurements (n = 8). Values are presented as means ± SEM. ‘NS’, not significant.

**Supplementary Fig. 3** Localization and action of HIF-2α during osteoblast differentiation. **a** Nuclear and cytoplasmic fractions were prepared in the absence or presence of osteogenic differentiation medium (DM). HIF-2α protein levels were examined by western blotting and quantified by ImageJ (n = 4). **b** Detection of HIF-2α in differentiated osteoblasts on day 6 of differentiation culture, as assessed by immunofluorescence microscopy. Nuclear-localized HIF-2α were counted in the indicated compartments of images (n = 10; scale bar, 100 μm). **c** Alkaline phosphatase (ALP) and alizarin red S (ARS) staining in primary calvarial osteoblasts infected with 400 MOI of control virus (Ad-C) or the indicated MOI of Ad*-Hif-2α* (n = 3). **d, e** Protein levels of HIF-2α were detected by western blotting in primary cultured calvarial pre-osteoblasts infected with 400 MOI of Ad-C or the indicated MOI of Ad*-Hif-2a* (n = 4, **d**), and transfected with control siRNA (si-C) or the indicated amounts (nM) of *Hif-2α*-siRNA (n = 4, **e**). Values are presented as means ± SEM (**P* < 0.05 and ****P* < 0.005).

**Supplementary Fig. 4** HIF-2α blocks osteoblast differentiation by upregulating TWIST2 expression. BMP-2-induced bone regeneration was measured in calvarial bone from WT and *Hif-2α*^+/-^ mice. Representative µCT images and measurement of bone volume of calvarial defect models applied with Ad-C or Ad-sh*Twist2* (n = 5). Values are presented as means ± SEM (**P* < 0.05).

**Supplementary Fig. 5** Osteoblast-specific depletion of HIF-2α increases bone mass. **a.** Double staining of HIF-2α and OCN was conducted by immunofluorescence microscopy (n = 7; scale bar, 10 μm). Nuclei were detected by DAPI (4’,6-diamidino-2-phenylindole). **b**. Representative images of H&E and TRAP staining in OVX- or sham-operated 3-month-old *Hif-2α*^fl/fl^ and *Hif-2α*^fl/fl^;*Col1a1-Cre* mice (n = 8; scale bar, 100 μm). BV/TV, N.Ob/B.Pm, Ob.S/BS, N.Oc/B.Pm and Oc.S/BS were obtained from bone histomorphometric analyses of the metaphyseal regions of femurs. **c, d**. Transcript levels of *Vegf* and the number of CD31-positive blood vessels were determined by qRT-PCR (n = 4; **c**) and immunofluorescence staining (n = 9; **d**) (scale bar: 100 μm) . Values are presented as means ± SEM (* *P*<0.05; ***P*<0.01; ****P*<0.005). The effects of OVX and osteoblast-specific deletion of *Hif-2α* as well as their interaction in mice were analyzed by two-way ANOVA (**a**, BV/TV: interaction = 0.0024, OVX < 0.0001, cKO < 0.0001)

**Supplementary Fig. 6** Verification that ZINC04179524 inhibits HIF-2α activity. **a** Primary chondrocytes transfected with 4xHRE-luc and pCMV-β-gal were treated with 2 ng/ml IL-1β in the presence or absence of ZINC04179524 (n = 4). **b** Transfected cells were infected with Ad-C or Ad-*Hif-2α* (800 MOI) and treated with or without ZINC04179524 (n = 4). Luciferase activities were normalized with respect to the corresponding β-gal activities. Values are presented as means ± SEM (*** *P*<0.005).

**Supplementary Fig. 7** Osteoclast-specific depletion of HIF-2α increases bone mass. **a**. Co-localization of HIF-2α and CTSK was determined by double immunostaining and quantified by measuring fluorescence intensity against HIF-2α in CTSK-positive osteoclasts (n = 8; scale bar, 10 μm). **b** Representative images of H&E staining, TRAP staining and bone histomorphometric analyses in OVX or sham-operated 3-month-old *Hif-2α*^fl/fl^ or *Hif-2α*^fl/fl^;*Ctsk-Cre* mice (n = 8; scale bar, 100 μm). Values are presented as means ± SEM (* *P*<0.05; ** *P*<0.01; *** *P*<0.005). ‘NS’, not significant. The effects of OVX and osteoclast-specific deletion of *Hif-2α* (cKO) as well as their interaction in mice were analyzed by two-way ANOVA (**a**, BV/TV: interaction = 0.0481, OVX < 0.0001, cKO = 0.0001)

**Supplementary Table 1.** PCR primers and conditions

| Gene | Strand | Sequences (5’-3’) | Size (bp) | ^a^AT  (℃) | Origin |
| --- | --- | --- | --- | --- | --- |
| *Ctsk* | ^b^S | TGTGGGTGTTCAAGTTTCTGCTGCTAC | 360 | 60 | Mo |
|  | ^c^As | TAGTCGATGGAGTCTGGGACCCTG |  |  |  |
| *Dcstamp* | S | TTGTGGCTGGAAGTATGAGAATGTC | 341 | 60 | Mo |
|  | As | ACGACTCCTTGGGTTCCTTGCTTC |  |  |  |
| *Runx2* | S | GCCACCTTTACCTACACCCC | 363 | 55 | Mo |
|  | As | GACTCATCCATTCTGCCGCT |  |  |  |
| *Hif-1α* | S | AGGCTCACCATCAGTTATTTACGTGTG | 421 | 60 | Mo |
|  | As | TAGACATGAATATGGCCCGTGCAGTG |  |  |  |
| *Hif-2α* | S | AGAAGAGCAAAGACGTGTCCACCGAG | 347 | 63 | Mo |
|  | As | GTAGAACTCATAGGCAGAGCGTCCAAG |  |  |  |
| *Nfatc1* | S | ACCACTCCACCCACTTCTGACTTC | 349 | 58 | Mo |
|  | As | AGCTGTAGCGTGAGAGGTTCATTC |  |  |  |
| *Ocn* | S | CTCCTGAGAGTCTGACAAAGCCTT | 320 | 55 | Mo |
|  | As | GCTGTGACATCCATTACTTGC |  |  |  |
| *Ocstamp* | S | CTCTCTCTGTGGTCTCTTCGTCTTC | 343 | 60 | Mo |
|  | As | TGTGAAGGCGGAAGGCTGAG |  |  |  |
| *Rankl* | S | GACTCGACTCTGGAGAGTGAAGAC | 353 | 60 | Mo |
|  | As | AATGTTGGCGTACAGGTAATAGAAG |  |  |  |
| *Traf6* | S | GCAGTGAAAGATGACAGCG | 255 | 59 | Mo |
|  | As | TTTCCAGCAGTATTTCATTG |  |  |  |
| *Trap* | S | CACGATGCCAGCGACAAGAGGTTC | 366 | 58 | Mo |
|  | As | AAACGTAGTCCTCCTTGGCTGCTGC |  |  |  |
| *Twist1* | S | TGAGCAACAGCGAGGAGGAG | 417 | 60 | Mo |
|  | As | GCCAGTTTGAGGGTCTGAATC |  |  |  |
| *Twist2* | S | GGCCGCCAGGTACATAGAC | 103 | 60 | Mo |
|  | As | GTAGCTGAGACGCTCGAGA |  |  |  |
| *Traf6*  #1 ChIP | S | GCCTCTTGCTAAGTTCTCCCAC | 109 | 58 | Mo |
|  | As | AAGAAAAGGCAGGGCATTGGA |  |  |  |
| *Traf 6*  #2 ChIP | S | CTAACTTGCTCCAGAGGTCACA | 204 | 58 | Mo |
|  | As | CTATTGCCAAAAGTTGTCCTCTGA |  |  |  |
| *Traf6*  #3 ChIP | S | CGTGACAATGTTGGAGAATGGG | 174 | 58 | Mo |
|  | As | TCCACGCTGAAGCCTTACCC |  |  |  |
| *Twist2*  #1 ChIP | S | TGAGAATGTTTAAGGAGCCA | 150 | 60 | Mo |
|  | As | GCTGTGTATGCCACTTCTTG |  |  |  |
| *Twist2*  #2 ChIP | S | CCAACACTCAGGGCAAGGTC | 182 | 60 | Mo |
|  | As | CTCGGGCCAGTATGCAAGTTG |  |  |  |
| *Rankl*  ChIP | S | AAAGGCACTTGGGAGGGAGTTCTAG | 123 | 60 | Mo |
|  | As | AAATCTTAGAGGAGACCACCATCAAG |  |  |  |

^a^AT,annealing temperature; ^b^S,sense primer; ^c^As,antisense primer
